# Change in psychosocial factors connected to coping after inpatient treatment for substance use disorder: a systematic review

**DOI:** 10.1186/s13011-019-0210-9

**Published:** 2019-05-03

**Authors:** Dagny Adriaenssen Johannessen, Trond Nordfjærn, Amy Østertun Geirdal

**Affiliations:** 1 Blue Cross East, Oslo, Norway; 2 Department of Psychology, Norwegian University of Science and Technology (NTNU), Trondheim, Norway; 3 Faculty of Social Sciences, Department of Social Work, Child Welfare and Social Policy, OsloMet – Oslo Metropolitan University, Pb 4, St. Olavs plass, 0130 Oslo, Norway

**Keywords:** Substance-related disorders, Residential treatment, Treatment outcome, Follow-up study, Social adjustment, Recovery capital

## Abstract

**Background:**

Among the adult population worldwide, about 0.5% has illicit drug use disorder (DUD) and about 5% has alcohol use disorder (AUD). Dependency on alcohol, medication or illicit drugs are recognised as risk factors for disabling disease and early death. Treatment for substance use disorders (SUD) is important in promoting persistent abstinence and may be perceived as a valuable public health measure. The current systematic review aims at exploring how psychosocial factors connected to recovery capital and coping behaviour, change after inpatient SUD treatment.

**Methods:**

A systematic search was conducted in Campbell Collaboration Library, Cochrane Library, EMBASE, Epistemonikos, Medline, PsychINFO, Social Sciences Citation Index and SocINDEX. Cohort studies on psychosocial outcomes for adults who had attended to inpatient SUD treatment that exceeds 3 months, were included. The outcome of interest was change in psychosocial factors. The search results were identified as *include*, *exclude* or *unclear* by one author and then screened by the second author with a specific focus on studies recognised as unclear. Diverging evaluations of eligibility among the *unclear* studies were resolved by discussion. In case of disagreement, the third author decided the eligibility of the studies in question.

**Results:**

Findings imply an overall progress in mental health, and a potential improvement in employment status and perceived social support after inpatient SUD treatment. Additionally, findings indicate a decrease in substance use from admission to follow-up after discharge from inpatient SUD treatment. These findings are consistent with earlier research on important factors in recovering from SUD. Findings on change in self-efficacy, housing, education and Quality of Life (QoL) however, were scantly researched and were expected to be more prominent outcomes of interest among the included studies.

**Conclusion:**

Due to the substantial resources used to provide SUD treatment, knowledge about recovery capital, like psychosocial factors that facilitate coping behaviour and reintegration to society, should be standardised and used by SUD treatment providers.

**Trial Registration:**

PROSPERO registration ID: CRD42018087408

**Electronic supplementary material:**

The online version of this article (10.1186/s13011-019-0210-9) contains supplementary material, which is available to authorized users.

## Background

About 5 % of the adult population worldwide have used illicit drugs at least once within the previous year. Approximately 10 % of these individuals have drug use disorder [1]. Records from 2014 reveal that more than 200.000 people suffered a drug-related early death worldwide that year, [2] a number which simply is too high. Additionally, half of the world’s adult population have consumed alcohol during the prior year, and more than 5 % of the world’s population have alcohol use disorder. Alcohol consumption is identified as one of the leading risk factors for disabling disease and shortening of life and represent one of the most prevalent causes of death worldwide [3]. On this background treatment for *substance use disorder* (SUD) may be perceived as a valuable public health measure [1].

Substantial resources are used internationally to provide treatment for SUD, substitution treatment and to treat SUD-related health problems [4]. Still, it is estimated that more than half relapse to substance use in the time after discharge from SUD treatment [5–7], even if relapse does not necessarily mean that the individual proceeds to use the same amount(s) of substance(s) as pre-treatment [8]. In *the 10th edition of the International Statistical Classification of Diseases and Related Health Problems* (ICD-10), SUD is described as “a cluster of physiological, behavioural, and cognitive phenomena in which the use of a substance or a class of substances takes on a much higher priority for a given individual than other behaviours that once had greater value” (p. 75) [9].

Some scholars have advised against comparing different treatment modalities with each other [10], and conclude that change in psychosocial function tends to occur regardless of modality [11, 12]. For instance, findings from extensive prospective cohort studies of patient outcome after SUD treatment such as the *Drug Abuse Treatment Outcome Study* (DATOS), *Treatment Outcome Prospective Study* (TOPS) and *National Treatment Outcome Research Study* (NTORS), show a clear reduction of substance use after completed treatment [10, 13–18]. Inpatient SUD treatment is considered to be important in promoting persistent abstinence [19] as well as personal, social and environmental change [1].

Much is known about psychosocial factors that facilitates recovery capital and increase the ability to cope in everyday life without substance use. Here, psychosocial factors refer to aspects which are currently conceptualized within the term recovery capital, like social support, housing, meaningful activity (e.g. employment and education), mental health and Quality of Life (QoL). Recovery capital can be described as individual or environmental attributes which facilitate the ability to recover from SUD, such as coping behaviour, and personal, structural and social resources [20–23]. Coping behaviour, which is embraced by recovery capital, is understood in line with Neale [24], as “managing negative feelings and bodily changes rather than trying to prevent them from occurring” (p. 32). Coping, thus, involves responses or actions taken in a given situation, or, coping behaviour [25]. Examples of facilitating psychosocial factors are social support and housing [24, 26–31], employment and education [6, 24, 26–28, 30, 32], treatment completion and commitment to continued care discharge plans [27, 32, 33]. However, these findings are mostly related to recovering from substance abuse after outpatient treatment, inpatient treatment with a duration of less than 3 months (short-term), peer-support or no treatment, while we know less about change in psychosocial factors after inpatient SUD treatment exceeding 3 months (long-term). Even if long-term inpatient SUD treatment is uncommon in some parts of the world, previous research suggests a better treatment outcome related to protective psychosocial factors, behaviour and maintained abstinence from substance use when comparing short-term with long-term SUD treatment [16, 34–38]. Additionally, long-term inpatient SUD treatment has been recognised as one of the most common modalities [39, 40], and has traditionally been included in extensive treatment outcome studies in the United States and Europe (see e.g. [10, 41–44]). We know less about SUD treatment duration in developing countries, even though SUD appears to be a widespread issue of concern [45].

To our knowledge, there are no systematic reviews to date that have aimed at exploring how psychosocial factors connected to recovery capital, change after long-term inpatient SUD treatment. This study originally aimed at exploring psychosocial factors associated with coping after inpatient SUD treatment. During the analysis process, however, we discovered that the findings also could provide information about *change* in psychosocial factors after inpatient SUD treatment. Therefore, the current systematic review aims to use findings from the systematic search to explore the following research question:

*How do psychosocial factors connected to coping, change after inpatient treatment for substance use disorder?*

## Methods

The overarching objective of a systematic review is to gather, unify and summarise research findings in the purpose to synthesise a new body of knowledge [46]. The current systematic review takes form as a thematic summary and purposes to outline overall findings by exploring differences and similarities among findings from the included studies. The systematic search bases on more or less pre-defined concepts except the sought outcome, namely the psychosocial factors. The search was designed with the purpose of allowing multifarious psychosocial factors to emerge among the results. The reason for this broad approach was that we wanted to also reveal studies about psychosocial factors that potentially could contribute to elucidate the current aim. The purpose of this review is to generate a body of knowledge about change in psychosocial factors enabling coping behaviour in everyday life after inpatient SUD treatment. Findings will be structured and presented in line with characteristics of the extracted findings from the included study reports.

### Search strategy

A set of text words (see Additional file 1) was developed by determining the *Population*, *Exposure* and *Outcome* (PEO) [47] of interest, and by exploring definitions, keywords, and indexing of related studies. Based on the included text words, subject headings (see Additional file 2) were identified in each of the included databases, which will be outlined below. Together, the combination of text words and the database-specific subject headings constitute the search string (see Additional file 3) used in the systematic search.

**Fig. 1:**
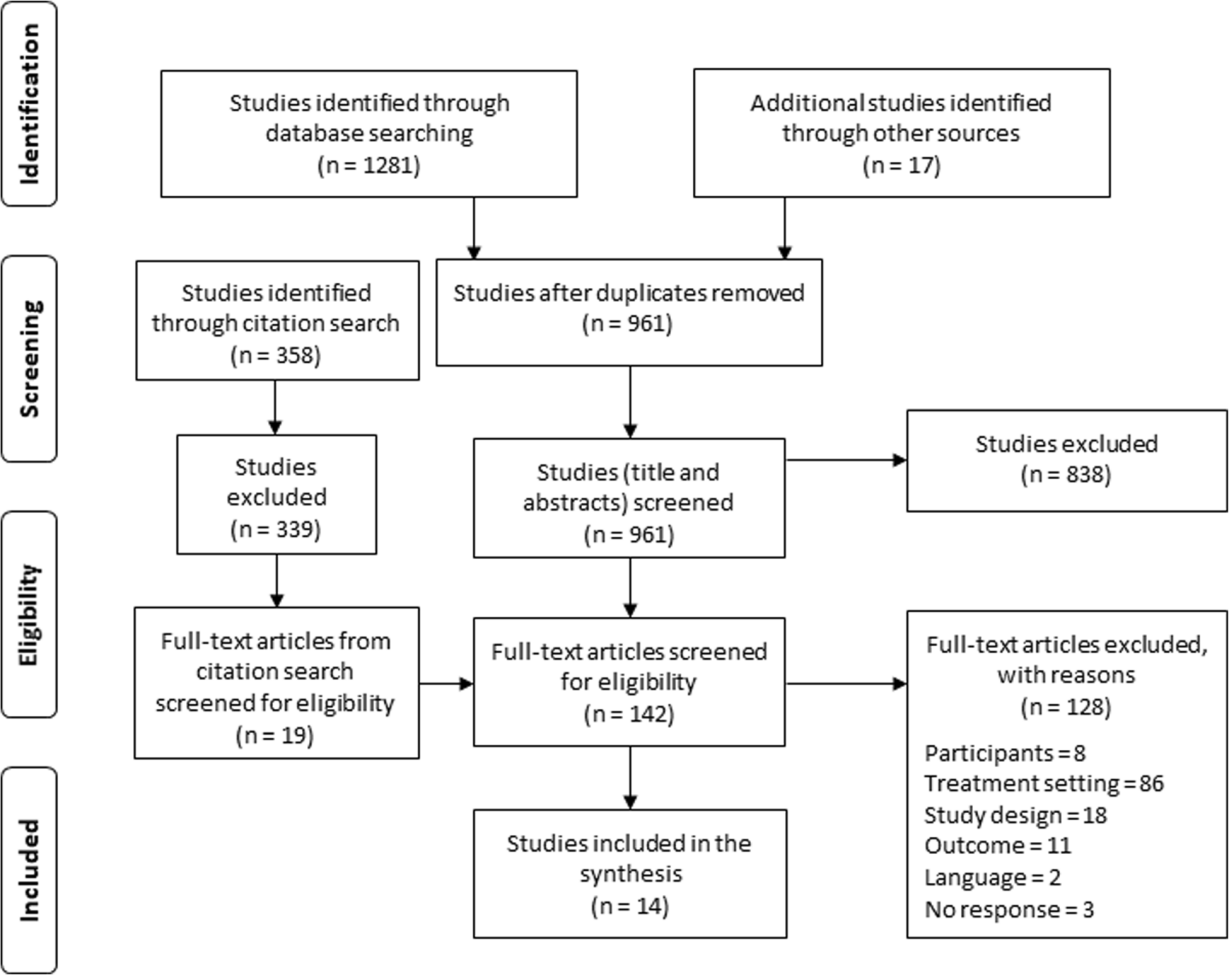
PRISMA Flow Diagram [69]

The aim of this review is situated in an intersection point between two research fields; medicine/health and social sciences. Therefore, and because the chosen databases should reflect research that is desirable to explore the aim of interest, the selected databases have their main focus in the field of medicine/health and/or social sciences. A comprehensive search was conducted in Campbell Collaboration Library, Cochrane Library, EMBASE, Epistemonikos, Medline, PsychINFO, Social Sciences Citation Index and SocINDEX. To increase the probability of detecting eligible studies that were not embraced by these databases, a citation search was undertaken in Web of Science. Additional screening for eligible studies was performed within the first 100 hits from an advanced search, using the established set of text words, in Google Scholar (reported as other sources in Fig. 1). To ensure a satisfying quality, the current review has been conducted and reported according to the *Preferred Reporting Items for Systematic Reviews and Meta-Analyses* (PRISMA) [48].

### Procedure

The search results were combined and controlled for duplicates using EndNote X8. One author (DAJ) eliminated irrelevant studies by screening titles and abstracts. After the first screening, the remaining studies were identified as *include*, *exclude* or *unclear* by the same author (DAJ)*,* and then screened by the second author (TN) with a specific focus on studies which were recognised as unclear. Diverging evaluations of eligibility among the *unclear* studies were resolved by discussion. In case of disagreement, the third author (AØG) decided the eligibility of the *unclear* studies.

The authors developed and pre-piloted a form to extract data of interest from the eligible studies. The relevance of the included studies was evaluated by one author (DAJ), using the *Critical Appraisal Skills Programme* (CASP) [49] 12-point checklist tool, developed to evaluate quality and relevance of included cohort studies. CASP is not recommended as a tool to report the quality-score [49] and has therefore not been further included in the results or discussion.

In cases where an eligible study did not report the data of interest to the current review, the corresponding author was contacted to obtain their opinion. This became applicable in four studies [50–53]. Three of the corresponding authors did not respond within 14 days, and the third author (AØG) determined the eligibility of these studies.

### Primary and secondary outcome

The outcome of interest was change in psychosocial factors (e.g. social support, employment, mental health or QoL) after inpatient SUD treatment. Measures revealing prevalence of continued substance use after discharge are considered as the secondary outcome of interest. The reason for this was twofold and partly because the SUD population that are referred to inpatient treatment often strives with multiple psychosocial challenges, comorbidity and to handle everyday life [54–57], and rarely only strives with dependency on substances. Change in psychosocial factors after inpatient SUD treatment has been acknowledged as a valid measure for treatment outcome [1], which also was an important reason for primarily focusing on psychosocial factors.

### Inclusion criteria

The results from the systematic search were screened and evaluated for eligibility based on the following pre-determined criteria. **Population:** adult men and women who were 18 years or older. **Exposure:** inpatient SUD treatment with a treatment duration exeeding 3 months. **Outcome:** various psychosocial factors. **Study design:** cohort studies with one or more follow-up measures after discharge from the index treatment. **Reporting:** peer reviewed study reports published in English. The systematic search was conducted without specific timespan-restrictions.

The reason for excluding studies exploring short-term inpatient treatment was the considerable resources used to provide SUD inpatient treatment [4], and that previous research has shown a positive correlation between long-term treatment and a favourable treatment outcome [16, 58]. This correlation can, however, occur as a result of the extended treatment duration and the opportunity this gives for more detailed observations. The reason for excluding outpatient treatment was to reach studies with a SUD population that strives with multiple psychosocial challenges and comorbidity, and who struggles to handle everyday life (e.g. attending appointments, living at home, attend to work or activity, maintain daily routines), which often is the case for those who are referred to inpatient treatment [54–57]. Additionally, previous systematic reviews and meta-analyses have already summarised effect-studies of various outpatient treatments and services [59–63]. Inpatient SUD treatment is considered to be important in promoting persistent abstinence [19] as well as personal, social and environmental change [1]. Inpatient SUD treatment can be described as interventions and measures aiming to alter conditions leading to destructive conditions and corroborate behaviour that reduces problematic substance use, which takes place at a treatment facility where the patient is a resident [64].

A comparison or control group was not a methodological requirement for inclusion in this review. A considerable degree of the treatment outcome research in the SUD field are observational naturalistic studies (see e.g. [10, 17, 36, 41, 42, 65]). Even if comparing pre- and post-treatment measures involves some pitfalls, a randomised controlled study design is not necessarily a viable option when studying change in multifarious psychosocial factors after treatment [66–68]. Based on the mentioned issues, and as further narrowing of the eligibility criteria could reduce the probability of detecting studies that serve the mentioned purpose, we decided to include both studies with and without a comparison.

### Ethical statement

The current target group, people with SUD, might be considered as vulnerable. On this background, this population was the group of interest a review of already conducted studies. Patients have not been involved in this study and an ethical approval has therefore not been sought.

The study was registered in PROSPERO (registration ID: CRD42018087408) in the planning stage and has been updated consecutively.

## Results

### Identification

The selection steps are presented in the PRISMA Flow Diagram [69] (see Fig. 1), where the listed inclusion criteria are arranged in the same order as reason for exclusion (1) participants, 2) setting, 3) study design, and 4) outcome). One thousand two hundred eighty-one studies were identified through the systematic search. Nine hundred forty-four studies remained after the duplicates were removed. Additionally, 375 studies were identified through Google Scholar and citation searches. In total, 1319 titles and abstracts were screened, whereupon the eligibility of 1177 (838 studies from the main search and 339 studies from the citation search) studies was determined. The remaining 142 studies were read in full-text. Finally, 14 studies were found to be eligible for inclusion. See Fig. 1 for a detailed list of reasons for exclusion after the full-text read through.

### Characteristics

Characteristics of the included studies are summarised in Table 1. Findings related to change in psychosocial factors after inpatient SUD treatment, are presented in Table 2.

**Table 1:** Characteristics of the included studies

Author(s)	Country	Data period	Sample	Treatment duration	N at base-line	N at follow-up	Loss to follow-up	Follow-up interval	Study details
Andersen (2018) [73]	USA	1999–2000	Imprisoned women with SUD	> 6 months	119	101	16%	12-months post releas	The study examined the effect of social support on treatment outcomes for incarcerated women participating in a SUD treatment programs, which were compared with a lower intensity-program. The experimental program was eligible for inclusion in this review (56% of the total number of participants).
Burling et al. (1994) [78]	USA	1988–1989	Homeless people with SUD	> 100 days	110	79	29%	3, 6, 9, and 12- months post-discharge	The study examined change in factors like housing, employment, substance use and social relations after completed residential SUD treatment for homeless veterans.
Cuskey et al. (1979) [70]	USA	1974–1977	Women with SUD	> 6 months	222	97	56%	6- and 12- months post-discharge	The study described the status at follow-up regarding different psychosocial factors (e.g. criminal activity, employment, substance use) after participation in a residential SUD treatment program for mothers with their children. 31% of the participants had stayed in treatment for more than 3 months and was therefore eligible for inclusion in this review.
Donovan et al. (2001) [72]	USA	1996–1998	Veterans with co-occurring disorders	> 12 weeks	N/A	46	N/A	6- and 12-months post-discharge	The study examined change in Post Traumatic Stress Disorder (PTSD) symptoms and substance use after participation in SUD treatment for veterans with co-occurring disorders.
Flora & Stalikas (2012) [71]	Greece	2008–2009	People with SUD	> 6 months	157	50	68%	3-months post-discharge	The study explored change in important factors (e.g. mental health, self-efficacy, social support) among patients who had undergone SUD treatment.
Grella & Shi (2011) [76]	USA	1999–2002	People with co-occurring disorders	> 90 days	400	310	23%	6- and 12-months post-admission	The study examined the connection between treatment duration and different psychosocial factors (e.g. psychological distress, arrest) at follow-up in patients with co-occurring disorder undergoing SUD treatment.
Hubbard et al. (2003) [16]	USA	1991–1993	People with SUD	> 3 months	N/A	331	N/A	1- and 5-years post-treatment	The study evaluated the effect on substance use and psychosocial factors in four different SUD treatment modalities. Data from the Treatment Outcome Prospective Study (TOPS) and the Drug Abuse Treatment Outcome Studies (DATOS) cohorts were compared. One of the modalities was eligible for inclusion in this review (24% of the total number of participants).
Ludwig et al. (2013) [77]	Switzerland	–	People with SUD	> 4 months	805	415	49%	1-year post-discharge	The study examined psychosocial predictive factors (e.g. mental health, self-efficacy) on substance use at follow-up after SUD treatment.
McGuire et al. (2011) [74]	USA	2002–2005	Homeless veterans with SUD	> 90 days	840	640	24%	1-, 3-, 6- and 12-months post-discharge	The study examined the effect of three SUD and/or psychiatric treatment modalities on psychosocial factors (e.g. employment, housing). Two of the modalities were eligible for inclusion in this review (64% of the total number of participants).
Porowski et al. (2004) [79]	–	1996–2001	Women with SUD	> 6 months	1798	1181	34%	6-months post-discharge	The study summarized pre-post change in psychosocial factors (e.g. mental health, employment, criminal activity, education) and substance use after participation in SUD treatment for women. Participants were recruited from 32 granted projects for woman with SUD.
Soyez et al. (2006) [75]	Belgium	2000–2002	People with SUD	> 12 months	203	124	39%	12 to 18-months post-discharge	The study investigated the effect of social support in SUD treatment on psychosocial factors (e.g. mental health, employment, criminal activity) and substance use. In the study, one experimental group (32% of the total sample) was compared with a control group. The experimental group received a social network intervention in addition to treatment as usual, while the control group received treatment as usual. Both the experiment- and the control group were eligible for inclusion to this review.
Sung & Chu (2011) [83]	USA	1992–1995	Probatio-ners and parolees with SUD	> 6 months	1147	296	74%	12-months post-discharge	The study examined change in employment at follow-up after four different modalities of SUD treatment for probationers and parolees. Data were extracted from the original DATOS cohort. One of the modalities was eligible for inclusion in this review (31% of the total number of participants).
Warren et al. (2007) [80]	USA	1999–2003	People with co-occurring disorders	> 90 days	400	351	12%	6-month post-discharge	The study examined the role of psychosocial factors (e.g. social support, self-efficacy) on mental health and substance use outcomes at follow-up after SUD treatment for patients with co-occurring disorders.
Zhang et al. (2003) [37]	USA	1993–1995	People with SUD	> 90 days	1183	653	45%	11-months post-discharge	The study investigated the relationship between treatment duration and substance use at follow-up after participation in SUD treatment. The data were collected from the TOPS cohort, which studied participants in four different treatment modalities. One of the modalities was eligible for inclusion in this review (30% of the total number of participants).

*NA* Not Applicable/Not Available

**Table 2:** Findings

Author(s)	Outcome	Findings	Secondary outcome
Andersen (2018) [73]	Social support	Emotional social support was measured with a self-report scale and was significantly (*p* < .001) related to abstinence at follow-up.	37% of the participants reported no use of illicit drugs in the time from release (discharge) to follow-up.
Burling et al. (1994) [78]	Depression	Self-reported prevalence of serious depression had decreased significantly (*p* < .001) from admission to follow-up.	63% of the participants reported not to have used any substances during the previous 30 days.
	Anxiety	Self-reported prevalence of serious anxiety had decreased significantly (*p* < .001) from admission to follow-up.	
	Social support	There was no self-reported change in numbers of close relationships from admission to follow-up.	
	Housing	The number of housed participants had increased with 89% from admission to follow-up. Participants were identified as housed if they did not report to have spent any nights outdoors, in shelters or abandoned buildings during the previous 3 months.	
	Employment	The number of employed participants had increased with 57% from admission to follow-up. Employment was identified as working full- or part time, attending to school, treatment or being retired or disabled most of the previous 3 months.	
Cuskey et al. (1979) [70]	Employment	17% (*n* = 5 of 30) was employed at follow-up.	83% (*n* = 25 of 30) was not using drugs at the time of follow-up. The data was obtained from participants (44%), friends and family (37%) and public support system (19%).
	Education	20% (*n* = 6 of 30) was involved in educational activity at follow-up.	
	Criminal activity	83% (*n* = 25 of 30) had not engaged in criminal activity at follow-up. The data were obtained from participants (44%), friends and family (37%) and public support system (19%).	
Donovan et al. (2001) [72]	Mental health	PTSD symptoms, which was measured with the Clinician-Administered PTSD scale (CAPS), had decreased significantly (*p* < .001 and Cohen’s d: 0.63) from pre-treatment to follow-up.	Days of alcohol use (Cohen’s d: 0.94), alcohol to intoxication (Cohen’s d: 0.81) and polysubstance use (Cohen’s d: 0.70) decreased significantly (*p* < .001) from pre-treatment to follow-up. Substance use was measured with the Addiction Severity Index (ASI).
Flora & Stalikas (2012) [71]	Depression	Depression was measured with the Depression Anxiety Stress Scale (DASS) and showed a statistically significant decrease (*p* < .000) from admission to follow-up.	N/A
	Anxiety	Anxiety was measured with DASS and showed a statistically significant decrease (*p* < .033) from admission to follow-up.	
	Negative emotions	Negative emotion was measured with a subscale accompanying the Differential Emotion Scale-Modified (DES-MOD) and showed a statistically significant decrease (*p* < .035) from admission to follow-up. Negative emotions were not defined in the current study, but refer to emotions like sadness, shame, remorse, embarrassment, fear or disgust.	
	Positive emotions	Positive emotion was measured with a subscale accompanying the DES-MOD and showed a statistically significant increase (*p* < .000) from admission to follow-up. Positive emotions were defined as emotions such as gratitude, love, pride, sympathy, peacefulness, hope and sexual energy.	
	Meaning of life	Meaning of life was measured with the Meaning in Life Questionnaire (MLQ) and had a statistically significant increase (*p* < .032) from admission to follow-up.	
	Social support	Social support was measured with the Multidimensional Scale of Perceived Social Support (MSPSS) and had a statistically significant increase (*p* < .000) from admission to follow-up.	
	Self-efficacy	Self-efficacy was measured with the Brief Situation Confidence Questionnaire (BSCQ) and had a statistically significant increase (*p* < .000) from admission to follow-up.	
Grella & Shi (2011) [76]	Mental health	Psychological distress (measured with the Brief Symptom Inventory) decreased (*p* < .0001 and Cohen’s d: 0.51) from admission to follow-up.	38% of the participants reported no alcohol or drug use the prior 6 months to follow-up.
	Criminal activity	Self-reported number of arrests decreased 71% from admission to follow-up.	
Hubbard et al. (2003) [16]	Criminal activity	Predatory illegal acts (assault, robbery, burglary, larceny, forgery, stolen property) decreased 24% from pre-admission to follow-up.	Participants reported to have modified their substance use from pre-admission to follow-up. 8% decrease in heroin use, 39% in cocaine use, 8% in marijuana use and 22% in alcohol use.
	Employment	Self-reported full-time employment among the participants increased 26% from pre-admission to follow-up, which was a significantly (*p* < .01) increase.	
Ludwig et al. (2013) [77]	Mental health	Psychological distress (measured with the Brief Symptom Inventory at admission was not a significantly predictor of abstinence at follow-up.	N/A
	Self-efficacy	General self-efficacy at admission, measured with one single question, was a significant (*p* < .05 and Confidence Interval (CI): 1.10 to 2.03) predictor of abstinence at follow-up. The abstinence rate was 28% higher in participants with a maximum general self-efficacy.	
McGuire et al. (2011) [74]	Housing	Housing increased 58% for the participants in the Domiciliary Care for Homeless Veteran Program (DCHV). Housing increased 61% for the participants in the Health Care for Homeless Veterans (HCHV) programme. Housing was determined by the housing-status (having a house or not) the night before the follow-up interview.	N/A
Porowski et al. (2004) [79]	Mental health	Even if participants who were abstinent at follow-up had significantly (*p* < .0001) less mental health problems than participants who had relapsed, both groups changed positively from pre- to post treatment. ‘Mental health problems’ were not further defined in the study report but has been defined by the World Health Organization as personal, environmental or social properties which facilitates a person’s well-being and ability to recover and contribute to and take part in society.	Self-reported abstinence from substance use throughout the 6-months following discharge increased significantly (*p* < .0001) from pre-admission to follow-up. 48% of the participants had not relapsed to substance use or had only used legal substances at follow-up. 61% of the participants had not used substances in the months prior to follow-up.
	Employment	Employment in the prior 30 days increased significantly (*p* < .0001) from pre-admission to follow-up for both groups.	
	Education	Employment increased significantly (*p* < .0001) from pre-admission to follow-up for both groups.	
	Criminal activity	Involvement in any criminal activity the past 30 days decreased significantly (*p* < .0001) from pre-admission to follow-up for both groups.	
Soyez et al. (2006) [75]	Mental Health	Psychological health increased significantly (*p* < .001) from admission to follow-up.	Use of alcohol and/or illegal drugs decreased significantly (*p* < .001) from admission to follow-up. Data were obtained using the European version of the Addiction Severity Index (EuropASI).
	Social support	Increased significantly (*p* < .001) from admission to follow-up.	
	Employment	There was no change in employment from admission to follow-up.	
	Criminal activity	Decreased significantly (*p* < .001) from admission to follow-up. Data were obtained using the European version of the Addiction Severity Index (EuropASI).	
Sung & Chu (2011) [83]	Employment	Participants who were unemployed at admission, had increased their employment rate by 28% at follow-up. ‘Employed’ participants were identified by self-reported employment-status (working at a legitimate job full- or part-time) the week before 12-month follow-up.	N/A
Warren et al. (2007) [80]	Mental health	Mental health was measured using the Research And Development Short Form-36 (RAND SF-36 scale). A positive relation (*p* < .001) was found between a high RAND SF-36 score and self-reported alcohol- or cocaine use at follow-up. Longer time in treatment predicted a lower RAND SF-36 score at follow-up.	Longer time in treatment predicted less alcohol use at follow-up. Substance use was identified as self-reported frequency of heroin-, cocaine/crack and/or alcohol use in the 30 days prior to follow-up.
Zhang et al. (2003) [37]		N/A	Self-reported days of overall drug use per month decreased (*p* < .001) from admission to follow-up. Self-reported days of primary drug use per month decreased (*p* < .001) from admission to follow-up.

N/A (Not Applicable/Not Available)

Mainly, the included studies were conducted in the United States (*n* = 10). However, Belgium (*n* = 1), Greece (*n* = 1) and Switzerland (*n* = 1) were represented as well. One of the studies did not report the country in which it was carried out. The systematic search sought for studies which were published until the time of the search (February 2018). However, findings revealed that data used in the eligible studies had been collected from 1974 [70] to 2009 [71], and the last point of follow-up ranged from 3 months to 5 years post-discharge. The total sample size at baseline was at least 7.384 participants. Two of the studies [16, 72] did not report the sample size at baseline. All the included studies reported sample size at follow-up after discharge, which was calculated to be a total of 4.674 participants. The mean (M) percentage of loss to follow-up among the 12 studies that reported sample size at both baseline and follow-up, was 39% (median (Mdn) = 37%). Primarily, the included studies had employed a pre-post cohort design. However, four studies [16, 73–75] used a comparative design where change in one group was compared to changes in another group that had received a different intervention. All the 14 included studies had a quantitative methodological orientation.

Unless specified otherwise, reported psychosocial change in Table 2 applies to the change from baseline to the *last* measure point at follow-up.

None of the studies reported effect sizes (Cohen‘s d) and few studies reported the M and standard deviation (SD) at baseline and follow-up. It was therefore not an option to include Cohen‘s d consistently when reporting the findings in this review. The authors of the current review calculated Cohen‘s d in studies that reported M and SD at both baseline and follow-up [72, 76]. These results are presented in Table 2. Only one study reported Confidence interval (CI) [77]. Accordingly, it was not viable to undertake and include a meta-analysis in this review. An overall statistical analysis has therefore not been provided in the findings presented hereunder.

In addition to what was defined as secondary outcome, potential change in six psychosocial factors related to recovery capital and coping behaviour were identified and will be described separately in the following sections.

### Mental health

The most studied psychosocial factor throughout the included studies was different versions of mental health (e.g. anxiety, depression, self-efficacy, psychological distress), which represented 15 outcomes in eight studies [71, 72, 75–80]. Mental health has been defined as personal, environmental or social properties which facilitates a person’s well-being and ability to recover and contribute to and take part in society [81]. Across the included studies, findings suggested a decrease in the following factors; depression and anxiety [71, 78], psychological distress [76, 77], PTSD-symptoms [72] and mental health problems [71, 79]. Findings further suggested an increase in the following factors; self-efficacy, perception of the meaning of life, positive emotion [71] and psychological health [75]. Additionally, one study [80] suggested that continued substance use was positively correlated with lower QoL at follow-up. Another study [77] suggested that self-efficacy at admission was predictive for abstinence at follow-up after inpatient SUD treatment. Self-efficacy has been recognised by Bandura [82] as a person’s confidence in its own ability to reach a goal.

Findings across these studies suggest an overall increase in mental health and a decrease in mental health problems from admission to follow-up after inpatient SUD treatment.

### Education and employment

Six studies reported findings related to change in employment status at follow-up after inpatient SUD treatment. Four studies [16, 70, 78, 83] presented change in percentage of which the M increase in employment rates from admission to follow-up after inpatient SUD treatment was 32% (Mdn = 27%). Furthermore, one study [79] suggested a significant increase in employment from admission to follow-up, while another study [75] reported no change in employment status from admission to discharge from inpatient SUD treatment.

In addition, education was the outcome of interest in two studies [70, 79]. Findings from one study [70] suggested that 20% of the participants were involved in educational activity at follow-up, while findings from the other study [79] suggested a significant increase in educational activity from pre-treatment to follow-up.

Findings across these studies suggest an overall increase in employment and a potential increase in educational activity from admission to follow-up after inpatient SUD treatment.

### Criminal activity

Five studies reported findings on engagement in criminal activity. Two of these studies [70, 76] reported the percentage of participants who had not engaged in any criminal activity from discharge to follow-up, which constitutes an M of 77%. Next, when comparing measures from pre-treatment to measures at follow-up, one study found that criminal activity had decreased with 24% [16] and another study found a significant reduction in criminal activity [75]. One study [79] reported a significant decrease in criminal activity during the 30 days prior to follow-up compared to pre-admission.

Findings across these studies suggest an overall decrease in criminal activity at follow-up after inpatient SUD treatment.

### Social support

Four studies reported findings related to social support. One of these [73] implied a correlation between abstinence and perceived social support at follow-up. One study [78] suggested that there was no change in close relationships from admission to follow-up after inpatient SUD treatment. Two studies [71, 75], however, reported a significant increase in social support from admission to follow-up. Social support is often operationalised as *instrumental* or *emotional*. Instrumental social support refers to properties in the support system like public services and financial aid, while emotional social support is identified as perceived interpersonal support and connectedness [84].

Findings across these studies suggest a possible, but slight, increase in perceived social support at follow-up after inpatient SUD treatment.

### Housing

Together with education, housing was the least studied psychosocial factor among the included studies. Housing represented the outcome of interest in two studies [74, 78]. The number of housed participants increased by an average of 69% from admission to follow-up.

Findings from these two studies suggest an increase in housed participants from admission to follow-up after inpatient SUD treatment.

### Secondary outcome

Four studies [71, 74, 77, 83] did not examine the prevalence of substance use at follow-up after inpatient SUD treatment. The remaining studies reported change in substance use from admission to follow-up. Findings from four studies [16, 37, 72, 75] suggested a decrease in substance use or an increase in abstinence at follow-up after inpatient SUD treatment. One study [79] reported both decreased substance use from admission to follow-up and the M percentage (55%) of abstinent participants at follow-up. Four studies [70, 73, 76, 78] showed that both the M and Mdn percentage of abstinent participants at follow-up was 55%. The last study [80] suggested a correlation between treatment duration and abstinence at follow-up after inpatient SUD treatment.

Findings from these studies suggest an overall decrease in substance use from admission to follow-up after inpatient SUD treatment.

## Discussion

The current report presents findings from a systematic review of studies that have explored change in psychosocial factors connected to recovery capital after inpatient SUD treatment. Findings from 14 eligible studies have been presented.

The findings imply an overall progress in mental health, and a potential improvement in employment status and perceived social support for people who undergo inpatient SUD treatment. Additionally, they indicate a decrease in substance use from admission to follow-up after inpatient SUD treatment. Even if pre- and post-measures related to lifetime prevalence of criminal activity and criminal activity between discharge and follow-up are non-comparable values, findings suggest a decreased engagement in criminal activity at follow-up compared to pre-admission. These findings are consistent with earlier research on factors that are important when recovering from SUD, such as meaningful activity [6, 24, 26–28, 30, 32], social support [24, 26–31] and mental health [85, 86].

Among the studies that took abstinence after discharge into consideration, the definitions varied. Abstinence at follow-up was defined as “no use of any substances at any time since discharge” (p. 196) by Porowski et al. [79]. Cuskey et al. [70] reported only findings related to the number of participants not using substances at follow-up, while Grella and Shi [76] explored substance use during the 6 months prior to follow-up. However, findings are in accordance with outcomes from previous comprehensive prospective studies showing a reduction in substance use after various modalities of SUD treatment [10, 13–15], as well as previous findings related to the proportion of participants who continue using substances after treatment [5–7].

Self-efficacy, housing, education and QoL were expected to be more prominent outcomes of interest among the included studies. Change in self-efficacy after inpatient SUD treatment was explored in only one study, Flora and Stalikas [71]. In addition, Ludwig [77] used measures of general self-efficacy to predict abstinence at follow-up and Sung and Chu [83] found self-efficacy to be a predictive factor for employment status after SUD treatment. On the other hand, previous research has identified self-efficacy as a key element related to coping with abstinence, adherence to treatment and successful recovery from SUD [50, 87, 88]. The cited studies examined changes in psychosocial factors after short-term inpatient SUD treatment as well as self-efficacy’s potential influence on personal change, such as recovering from SUD.

While earlier research findings have highlighted stable housing as an essential facilitator and motivation for wage labour and abstinence from substance use [28, 89, 90], just two studies [74, 78] presented housing status as the outcome of interest after inpatient SUD treatment. Thus, the insight that only two studies considered housed participants as a result of interest, did not correspond with the emphasis of housing as recovery capital, found in earlier research [24, 26–31]. Previous studies have examined different aspects of housing, including how people with SUD highlighted housing as an important factor in recovery and as an important motivation and part of continued care after different SUD treatment modalities.

Educational activity appears to be a scantly investigated outcome and was only explored in two of the included studies [70, 79]. However, according to Laudet and White [90], patients in all stages of recovery underline education as highly prioritised, but also as a subject of concern during their recovery. Employment seems to play an essential role in the motivation for recovering from SUD, as reported by Manuel et al. [28]. Further, education is considered as a valuable attribute in the labour market [91], also for people who have struggled with SUD [92]. As anticipated, Sung and Chu [83] found that education predicted personal income after SUD treatment. Sustainable personal income has in turn been emphasised as a motivation to afford basic properties in everyday life as abstinence [24, 28]. Previous research on the role of education in SUD recovery, were conducted in other contexts than inpatient treatment (e.g. outpatient, shot-term inpatient, continued care), and in connection to work and employment.

One study [74] explored how QoL was associated with a set of selected values but did not present separate results for participants adhering to long-term inpatient SUD treatment. Another study [80] examined how QoL was associated with treatment duration and continued substance use after inpatient SUD treatment. Previous research, however, suggests that SUD inpatients have lower QoL compared to the general population and SUD out-patients [93, 94]. QoL appears to improve after various interventions aiming to reducing substance use [94, 95], and decrease with stressful life events [95]. Additionally, research findings imply that SUD treatment facilitate reduced substance use [8, 10, 13–15], and improved everyday function [90], even if complete abstinence is not achieved. In view of the overarching international ambition to provide SUD treatment, relapse to substance use alone may be a weak standard of successful recovery. The negotiated objective of SUD treatment is to decrease the extent of substance use as much as possible, reduce the negative consequences for the individual, and to “improve function and well-being of the affected individual” (p. 7) [1]. Bearing this in mind, factors such as QoL and substance use after SUD treatment may be a more viable combination to measure successful recovery [94, 95]. On this background, QoL was expected to be more explored as an outcome of interest after inpatient SUD treatment, than the findings from this review revealed.

Finally, approximately half of the included studies were based on data obtained from vulnerable samples. Clinical diversity in such factors across individual studies stimulate to a discussion about potential impact on validity of the final syntheses. Vulnerable groups, like those included in the current review, oftentimes tend to be excluded from clinical research [96, 97] and may affect the treatment outcome in a negative manner [98]. Vulnerable subjects within an already vulnerable population, which the SUD population represents, are especially exposed and represents a minority in a subordinate group in society [1]. For instance, people with co-occurring disorders are afflicted with a severe mental health condition in addition to SUD, and people who lack a permanent home lack the basic needs which are recognised as fundamental in recovering from SUD [99]. Furthermore, women are underrepresented in the SUD population [100], which makes them an especially vulnerable group in an already exposed population [1]. Arguably the exclusion of such vulnerable groups from research may affect the representativity of the body of knowledge on the SUD field.

### Limitations

When interpreting findings presented in this review, it is important to take potential limitations into consideration. Even though all three authors have contributed throughout the planning of the procedure and the systematic search, only one author carried out the systematic search and the first screening of search results. This may serve as a gateway for selection bias, which may have influenced the final results. However, uncertainty related to choices was discussed between the authors inn all stages. The determined inclusion criteria, like the choice to only include long-term inpatient treatment or to exclusively consider studies that have applied a cohort study design, have most likely led relevant studies to be undetected in the systematic search or excluded during the screening. Still, the authors found it important to narrow the inclusion criteria to broaden the probability to find studies that could highlight the research question. Furthermore, there are aspects of the included studies that limit the opportunity to present clear-cut conclusions. For instance, across the included studies, data were mostly collected using self-reported measures. While this may serve as a limitation in some populations, findings from previous research reveal a good correspondence between self-reported substance use and biological samples like urine test or hair samples in the SUD population [16, 101–103] . Furthermore, in Porowski et al. [79] study, results concerning patient outcome were excluded from clinics that lost more than 50% of their participants to follow-up, an issue present in 18 out of 50 clinics. Additionally, Cuskey et al. [70] solely reported results from measures taken at follow-up. Finally, half of the included studies and approximately half of the participants in total, represents vulnerable groups. Even if clinical diversity is rather the rule than the exception when conducting an aggregative systematic review [104], the results and conclusions in this review are presented with caution and the findings should only be generalised if it is probable to assume that they are transferable to the current population.

## Conclusion

The current review has explored change in psychosocial factors connected to recovery capital and coping behaviour in everyday life after inpatient SUD treatment. Various factors like social support, meaningful activity (e.g. employment and education), criminal activity, mental health, psychological distress and mental health problems, have been previously studied. Earlier research emphasises the importance of factors connected to recovery capital, like self-efficacy, housing, education and QoL, which facilitates the ability to cope without substance use. Nonetheless, the present study indicates that knowledge about how inpatient SUD treatment influence the latter mentioned factors seems to be faint or absent and implies a need for more research on how these factors are associated with coping behaviour after inpatient SUD treatment.

Due to the substantial resources used to provide SUD treatment, knowledge about recovery capital, like psychosocial factors that facilitate coping behaviour and reintegration to society, should be standardised and used by SUD treatment providers. The current findings may, however, also encourage a critical view on how change in the mentioned psychosocial factors are connected to inpatient treatment in favour of other potentially influential factors.

## Additional files

Additional file 1: Text words used in the systematic search. (PDF 64 kb)

Additional file 2: Subject headings used in the systematic search in the included databases. (PDF 81 kb)

Additional file 3: Main search (example draft from the systematic search in Medline Ovid). (PDF 92 kb)

## Abbreviations

CASP: Critical Appraisal Skills Programme
CI: Confidence interval
DATOS: Drug Abuse Treatment Outcome Study
ICD-10: The 10th edition of the International Statistical Classification of Diseases and Related Health Problems
M: Mean
Mdn: Median
NTORS: National Treatment Outcome Research Study
PEcO: Population, exposure, outcome
PRISMA: Preferred Reporting Items for Systematic Reviews and Meta-Analyses
QoL: Quality of Life
SD: Standard deviation
SUD: Substance use disorder
TOPS: Treatment Outcome Prospective Study
