# Supplementary material for: Change in psychosocial factors connected to coping after inpatient treatment for substance use disorder: a systematic review
Source: Subst Abuse Treat Prev Policy. 2019 May 3;14:16. doi: 10.1186/s13011-019-0210-9 (PMC6499970; doi:10.1186/s13011-019-0210-9)
Supplement: Supplementary file 1 — Text words used in the systematic search. (PDF 64 kb) [file 13011_2019_210_MOESM1_ESM.pdf]

Supplementary material 1: Text words

P	("substance use disorder" OR "substance use" OR "substance abuse" OR "substance misuse" OR addict* OR dependency* OR alcoholism*)
	(narcotic* OR drug* OR alcohol OR heroin* OR opioid* OR cannabis* OR marihuana* OR cocaine* OR crack* OR amphetamine* OR methamphetamine* OR hallucinogen* OR morphine OR ecstasy* OR MDMA)
E(C)	(inpatient* OR resident* OR hospitali*)
	(treat* OR therap* OR rehab* OR recove*)
O	("psychosocial factors" OR "psychosocial aspects" OR "quality of life" OR "value of life" OR "mental health" OR mastery OR coping OR self-esteem OR "social wellbeing" OR "social support" OR "social capital" OR "material capital" OR "interpersonal relationship" OR employment OR education OR anxiety OR depression OR stigma)
SD	(cohort* OR longitudin* OR prospective* OR follow-up OR prognos* OR observational OR epedimiol*)
