# Supplementary material for: Change in psychosocial factors connected to coping after inpatient treatment for substance use disorder: a systematic review
Source: Subst Abuse Treat Prev Policy. 2019 May 3;14:16. doi: 10.1186/s13011-019-0210-9 (PMC6499970; doi:10.1186/s13011-019-0210-9)
Supplement: Supplementary file 2 — Subject headings used in the systematic search in the included databases. (PDF 81 kb) [file 13011_2019_210_MOESM2_ESM.pdf]

Supplementary material 2: Subject headings[1]

	Cochrane Library	Embase	Medline	PsychINFO	SocINDEX
P	Substance-Related Disorder	Drug dependence<br>Alcoholism<br>Substance abuse	Substance-Related Disorders	Alcohol Abuse<br>Drug Dependency<br>Polydrug Abuse<br>Substance Use Disorder	Substance abuse<br>Drug addiction<br>Alcoholism
E (C)	Residential Treatment	Drug dependence treatment	Inpatient<br>Residential Treatment	Residential Care Institution	Substance abuse treatment
O	Treatment Outcome<br>*AND*<br>Quality of Life<br>Value of Life<br>Mental health<br>Self Concept	Treatment outcome<br>Clinical outcome<br>Outcome assessment<br>Outcome research<br>Patient-reported outcome<br>*AND*<br>Social psychology<br>Stigma<br>Quality of life<br>Mental health<br>Self esteem<br>Self concept<br>Psychological well-being<br>Wellbeing<br>Social support<br>Social capital<br>Coping behavior	Treatment Outcome<br>*AND*<br>Quality of Life<br>Mental Health<br>Social capital<br>Social change<br>Social conditions<br>Social Environment<br>Value of life<br>Self Concept<br>Self Efficacy	Treatment Outcomes<br>Treatment Effectiveness Evaluation<br>*AND*<br>Psychosocial Factors<br>Quality of life<br>Well Being<br>Mental Health<br>Social Capital<br>Coping Behavior<br>Self-Efficacy<br>Self -Concept	Outcome assessment in psychotherapy<br>*AND*<br>Psychosocial factors<br>Quality of life<br>Mental health & social status<br>Social sustainability<br>Social adjustment<br>Self-esteem<br>Well-being

[1] In this table the two databases *Campbell Collaboration Library*, *Epistemonicos, Google Scholar* and *Social Science Citation Index* are excluded because the publications are not indexed regarding to subject headings.

SD	Cohort Studies	Cohort Analysis<br>Follow up	Cohort Studies	Cohort Analysis<br>Followup Studies	Cohort analysis
