# Supplementary material for: Change in psychosocial factors connected to coping after inpatient treatment for substance use disorder: a systematic review
Source: Subst Abuse Treat Prev Policy. 2019 May 3;14:16. doi: 10.1186/s13011-019-0210-9 (PMC6499970; doi:10.1186/s13011-019-0210-9)
Supplement: Supplementary file 3 — Main search (example draft from the systematic search in Medline Ovid). (PDF 92 kb) [file 13011_2019_210_MOESM3_ESM.pdf]

Supplementary material 3: Main search (search string used in Medline via Ovid, February 19th, 2018)

1 exp Substance-Related Disorders/

2 ("substance use disorder" or "substance use" or "substance abuse" or "substance misuse" or addict* or dependency* or alcoholism*).tw,kw,kf

3 1 or 2

4 (narcotic* or drug* or alcohol or heroin* or opioid* or cannabis* or marihuana* or cocaine* or crack* or amphetamine* or methamphetamine* or hallucinogen* or morphine or ecstasy* or MDMA).tw,kw,kf

5 3 and 4

6 exp Inpatients/

7 (inpatient* or resident* or hospitali*).tw,kw,kf

8 exp Residential Treatment/

9 6 or 7 or 8

10 (treat* or therap* or rehab* or recove*).tw,kw,kf

11 9 and 10

12 exp "Quality of Life"/

13 exp Mental Health/

14 exp Social Capital/

15 exp Social Change/

16 exp Social Conditions/

17 exp Social Environment/

18 exp "Value of Life"/

19 exp Self Concept/

20 exp Self Efficacy/

21 12 or 13 or 14 or 15 or 16 or 17 or 18 or 19 or 20

22 ("psychosocial factors" or "psychosocial aspects" or "quality of life" or "value of life" or "mental health" or mastery or coping or self-esteem or "social wellbeing" or "social support" or "social capital" or "material capital" or "interpersonal relationship" or employment or education or anxiety or depression or stigma).tw,kw,kf

23 21 or 22

24 exp Treatment Outcome/

25 23 and 24

26 exp Cohort Studies/

27 (cohort* or longitudin* or prospective* or follow-up or prognos* or observational or epedimiol*).tw,kw,kf

28 26 or 27

29 5 and 11 and 25 and 28
